# Surveillance for Lyme Disease — United States, 2008–2015

**DOI:** 10.15585/mmw.ss6622a1

**Published:** 2017-11-10

**Authors:** Amy M. Schwartz, Alison F. Hinckley, Paul S. Mead, Sarah A. Hook, Kiersten J. Kugeler

**Affiliations:** 1 Division of Vector-Borne Diseases, National Center for Emerging and Zoonotic Infectious Diseases, CDC

## Abstract

**Problem/Condition:**

Lyme disease is the most commonly reported vectorborne disease in the United States but is geographically focal. The majority of Lyme disease cases occur in the Northeast, mid-Atlantic, and upper Midwest regions. Lyme disease can cause varied clinical manifestations, including erythema migrans, arthritis, facial palsy, and carditis. Lyme disease occurs most commonly among children and older adults, with a slight predominance among males.

**Reporting Period:**

2008–2015.

**Description of System:**

Lyme disease has been a nationally notifiable condition in the United States since 1991. Possible Lyme disease cases are reported to local and state health departments by clinicians and laboratories. Health department staff conduct case investigations to classify cases according to the national surveillance case definition. Those that qualify as confirmed or probable cases of Lyme disease are reported to CDC through the National Notifiable Diseases Surveillance System. States with an average annual incidence during this reporting period of ≥10 confirmed Lyme disease cases per 100,000 population were classified as high incidence. States that share a border with those states or that are located between areas of high incidence were classified as neighboring states. All other states were classified as low incidence.

**Results:**

During 2008–2015, a total of 275,589 cases of Lyme disease were reported to CDC (208,834 confirmed and 66,755 probable). Although most cases continue to be reported from states with high incidence in the Northeast, mid-Atlantic, and upper Midwest regions, case counts in most of these states have remained stable or decreased during the reporting period. In contrast, case counts have increased in states that neighbor those with high incidence. Overall, demographic characteristics associated with confirmed cases were similar to those described previously, with a slight predominance among males and a bimodal age distribution with peaks among young children and older adults. Yet, among the subset of cases reported from states with low incidence, infection occurred more commonly among females and older adults. In addition, probable cases occurred more commonly among females and with a higher modal age than confirmed cases.

**Interpretation:**

Lyme disease continues to be the most commonly reported vectorborne disease in the United States. Although concentrated in historically high-incidence areas, the geographic distribution is expanding into neighboring states. The trend of stable to decreasing case counts in many states with high incidence could be a result of multiple factors, including actual stabilization of disease incidence or artifact due to modifications in reporting practices employed by some states to curtail the resource burden associated with Lyme disease surveillance.

**Public Health Action:**

This report highlights the continuing public health challenge of Lyme disease in states with high incidence and demonstrates its emergence in neighboring states that previously experienced few cases. Educational efforts should be directed accordingly to facilitate prevention, early diagnosis, and appropriate treatment. As Lyme disease emerges in neighboring states, clinical suspicion of Lyme disease in a patient should be based on local experience rather than incidence cutoffs used for surveillance purposes. A diagnosis of Lyme disease should be considered in patients with compatible clinical signs and a history of potential exposure to infected ticks, not only in states with high incidence but also in areas where Lyme disease is known to be emerging. These findings underscore the ongoing need to implement personal prevention practices routinely (e.g., application of insect repellent and inspection for and removal of ticks) and to develop other effective interventions.

## Introduction

Lyme disease, a tickborne zoonosis caused by spirochetes in the *Borrelia burgdorferi* sensu lato complex, can affect multiple human organ systems (*1*). *B. burgdorferi* sensu stricto is responsible for most infections in the United States, although *B. mayonii* also has been shown to cause human illness in the upper Midwest (*2*). Typical signs and symptoms in the days to weeks following a bite from an infected tick can include erythema migrans, fever, lymphadenopathy, arthralgia, myalgia, fatigue, and headache (*1**,**3**,**4*). The organism can infect the nervous system, causing facial palsy, and the cardiovascular system, causing carditis with atrioventricular heart block, a rare condition that can be fatal (*1**,**4**,**5*). Untreated infection might result in mono- or oligoarticular arthritis in large joints or, more rarely, encephalopathy and peripheral neuropathy (*4*). Patients with Lyme disease treated early with appropriate antibiotics usually experience a full recovery (*4*).

In the United States, human Lyme disease cases occur primarily in the Northeast, mid-Atlantic, and upper Midwest regions, but also in certain areas of the Pacific Coast (*6**,**7*). In all of these locations, competent vector ticks and infected reservoirs, such as small mammals and birds, are at sufficient density to support the enzootic cycle (*1**,**6**,**8*). The blacklegged tick, *Ixodes scapularis,* is the vector of Lyme disease in the eastern and upper midwestern United States; the western blacklegged tick, *I. pacificus*, is the vector of Lyme disease on the Pacific Coast (*1**,**6*). Commonly, larval blacklegged ticks infected during feeding transmit the bacteria to other hosts, including humans, during subsequent nymphal and adult stage blood meals (*1*).

Lyme disease has been a nationally notifiable condition in the United States since 1991 (*7**,**9*). Notable revisions to the case definition occurred in 1996 and 2008; the 2008 revision added reporting of cases meeting the probable case definition and narrowed the laboratory criteria for evidence of infection (*10*). The most recent summary of Lyme disease surveillance incorporated data collected during 1992–2006 (*7*). This report updates information acquired through national surveillance on the epidemiology of Lyme disease.

## Methods

### Data Source and Surveillance Case Definition

Public health agencies voluntarily transmit information on Lyme disease cases to CDC through the National Notifiable Diseases Surveillance System (NNDSS) (*11*). Variables transmitted include age, sex, race, ethnicity, and date of onset or of laboratory report. State health departments also can submit supplemental information on Lyme disease cases, including clinical manifestations of illness. Completeness of data specific to Lyme disease varies by state and over time. Cases are reported according to the patient’s state and county of residence rather than state and county of exposure. As a result, an infection could be acquired while visiting a state with high incidence but reported by a state with low incidence.

State and local health jurisdictions receive reports of potential Lyme disease cases from laboratories and clinicians. Per the surveillance case definition, laboratory reports require follow-up investigation to obtain clinical information necessary for appropriate case classification. As part of this system, states are responsible for classifying potential cases as confirmed or probable on the basis of criteria set forth in the case definition developed and approved by the Council of State and Territorial Epidemiologists (CSTE). Since 2008, a confirmed case of Lyme disease is defined as either 1) erythema migrans in a person who had possible exposure to tick habitat in an area where Lyme disease is endemic or who had laboratory evidence of infection or 2) at least one other defined clinical manifestation of Lyme disease in a person and laboratory evidence of infection (*10*). A probable case of Lyme disease is defined as laboratory evidence of infection in a person who had Lyme disease diagnosed by a clinician but with accompanying clinical information that does not meet the clinical criteria for a confirmed case. The 2008 surveillance case definition strengthened the specificity for laboratory evidence of infection. In the 2008 definition, sufficient laboratory evidence of infection for surveillance purposes was 1) a positive culture for *B. burgdorferi* and 2) a positive two-tier IgM or IgG serologic test (enzyme immunoassay followed by reflex immunoblot) interpreted using established criteria or a single-tier positive IgG immunoblot (*10*). Acute onset of specific neurologic, musculoskeletal, or cardiovascular signs and symptoms satisfy the criteria for clinical manifestations of confirmed Lyme disease (*10*). Beginning in 2011, the case definition reflected formatting changes in which text of the laboratory evidence of infection was modified to explicitly state that IgM two-tier serologic testing should only be interpreted in the first 30 days of illness onset, rather than solely referencing another document that outlined this criterion. Because the most recent surveillance summary (*7*) encompassed data collected during 1992–2006 and this report includes data collected following a notable case definition change in 2008 (e.g., reporting of probable cases and narrowing of laboratory criteria), data from 2007 are not included but are publicly available (*12*).

### Analysis

Data on Lyme disease cases reported to CDC during 2008–2015 were included. Annual incidence rates per 100,000 population were calculated by state using U.S. Census Bureau estimates from July 1 of each year (https://www.census.gov/). For this report, states were classified for surveillance purposes (state surveillance categories) as high incidence, low incidence, and neighboring. States with an average annual incidence during this reporting period of ≥10 confirmed Lyme disease cases per 100,000 population were classified as high incidence. States that share a border with those states or that are located between areas of high incidence were classified as neighboring states. All other states were classified as low incidence. Characteristics associated with cases reported from states in these three categories were compared. Percentage change in the number of reported cases between subsequent years was calculated for states with high incidence and neighboring states. The median annual percentage change in case counts over the reporting period was determined by selecting the median of each 1-year percentage change value for each state. Seasonality analysis was restricted to cases with illness onset dates no more than 1 year before the reporting year. Week of illness onset was calculated with each week beginning on Sunday. Week 1 of a year was the first week of the year that had at least 4 days in the calendar year; therefore, weeks 1 and 53 sometimes contained days from the preceding or subsequent year. Analysis of reported clinical signs and symptoms was restricted to only those records indicating at least one confirmatory sign or symptom and according to variables transmitted to CDC. All analyses were performed using SAS software version 9.3 (SAS Institute Inc., Cary, North Carolina).

## Results

**FIGURE 1:**
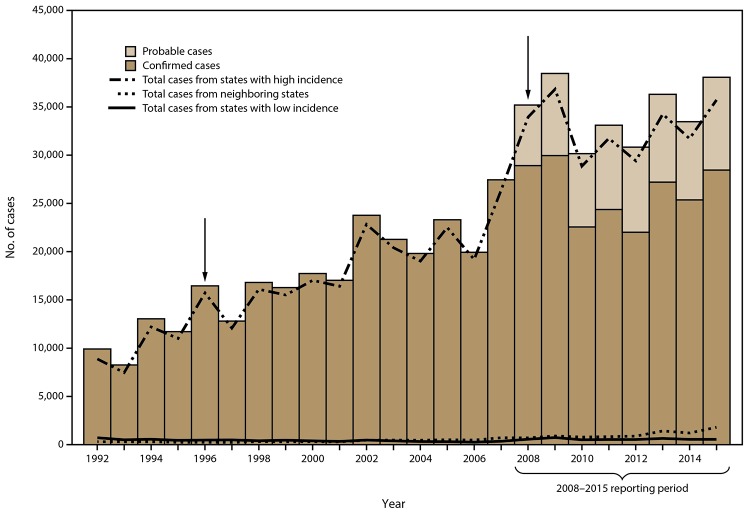
Number* of confirmed and probable Lyme disease cases, by state surveillance category^†^ and year — United States, 1992–2015^§^ * N = 551,107. ^†^ State surveillance categories were determined using three classifications: high incidence, neighboring, and low incidence. States with an average annual incidence ≥10 confirmed Lyme disease cases per 100,000 population were classified as high incidence, states that share a border with those states or are located between states with high incidence were classified as neighboring, and all other states were classified as low incidence. ^§^ Arrows indicate notable changes in case definitions. The case definition was revised in 1996 to recommend a two-step testing method and in 2008 to increase specificity of laboratory evidence of infection and to include provision for report of probable cases.

During 2008–2015, a total 275,589 cases of Lyme disease were reported to CDC (208,834 confirmed and 66,755 probable) (Figure 1). The combined annual total of confirmed and probable cases ranged from 38,468 in 2009 to 30,158 in 2010. The highest number of confirmed cases was reported in 2009 (29,959) and the lowest was reported in 2012 (22,014). Confirmed cases were reported from 48 states and the District of Columbia. On average, 8,344 probable cases were reported each year (range: 6,277 in 2008 to 9,616 in 2015). In 2008, a total of 35 states and the District of Columbia reported at least one probable case of Lyme disease; in 2015, a total of 41 states and the District of Columbia reported at least one probable case.

**FIGURE 2:**
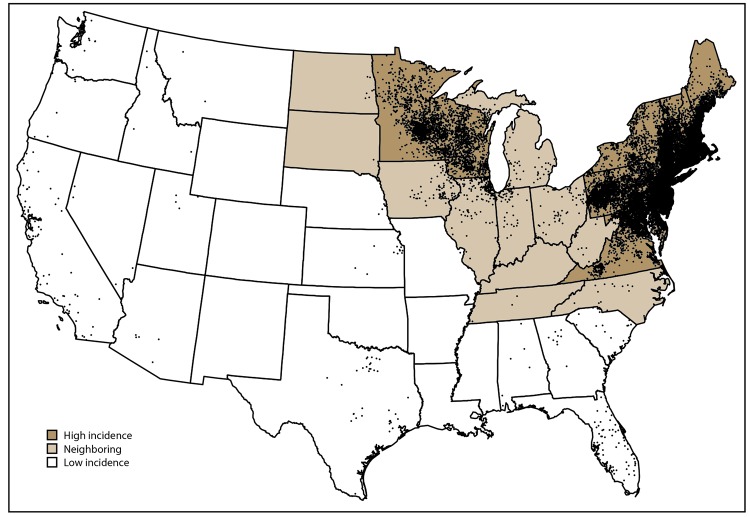
Average annual number of confirmed Lyme disease cases, by county of residence* — United States, 2008–2015^†^ * Each dot represents one confirmed case according to the patient’s county of residence. ^†^ State surveillance categories were determined using three classifications: high incidence, neighboring, and low incidence. States with an average annual incidence ≥10 confirmed Lyme disease cases per 100,000 population were classified as high incidence, states that share a border with those states or are located between states with high incidence were classified as neighboring, and all other states were classified as low incidence.

Fourteen states, all located in the Northeast, mid-Atlantic, and upper Midwest regions, met the criteria for classification as states with high incidence (Connecticut, Delaware, Maine, Maryland, Massachusetts, Minnesota, New Hampshire, New Jersey, New York, Pennsylvania, Rhode Island, Vermont, Virginia, and Wisconsin) (Table 1) (Figure 2). During 2008–2015, these states accounted for 95.2% of all reported cases and 95.7% of confirmed cases reported in the United States. Confirmed cases accounted for 76.2% of the total cases reported from states with high incidence. Despite the high number of reported cases, overall median annual percentage change in number of confirmed cases among these states was -0.29% (range: -18.8% to 20.1%). Seven of the 14 states displayed an overall decreasing trend in the number of confirmed cases, as indicated by negative median annual percentage changes.

**TABLE 1:** Annual rate* of confirmed Lyme disease, by state/area, state surveillance category,^†^ and year — United States, 2008–2015

State/Area	Year								Average
	2008	2009	2010	2011	2012	2013	2014	2015	
High incidence									
**Connecticut**	77.2	77.2	54.9	55.8	46.0	58.7	47.8	52.2	**58.7**
**Delaware**	87.3	110.3	72.9	84.5	55.3	43.2	36.4	35.3	**65.2**
**Maine**	58.6	59.5	42.1	60.3	66.6	84.8	87.9	74.7	**66.8**
**Maryland**	30.7	25.6	20.1	16.1	18.9	13.5	16.0	20.8	**20.1**
**Massachusetts**	61.2	61.7	36.3	27.2	51.0	56.9	54.0	43.0	**48.9**
**Minnesota**	19.9	20.1	24.3	22.2	16.9	26.4	16.4	21.4	**21.0**
**New Hampshire**	92.0	75.7	63.0	67.3	75.8	100.1	46.8	32.8	**69.1**
**New Jersey**	36.9	52.5	37.7	38.4	30.8	31.3	29.0	43.9	**37.5**
**New York**	29.9	21.4	12.3	16.0	10.4	17.8	14.4	16.4	**17.3**
**Pennsylvania**	30.3	39.1	25.9	37.2	32.5	39.0	50.6	57.4	**39.0**
**Rhode Island**	17.6	14.2	10.9	10.6	12.6	42.2	54.0	53.4	**27.0**
**Vermont**	52.9	51.7	43.3	76.0	61.6	107.5	70.5	78.4	**67.8**
**Virginia**	11.3	8.8	11.4	9.3	9.8	11.2	11.7	13.1	**10.8**
**Wisconsin**	26.5	34.4	44.0	42.2	23.9	25.2	17.2	22.7	**29.5**
Neighboring									
**District of Columbia**	12.2	8.9	5.6	N/A	N/A	5.1	5.3	11.6	**8.1**
**Illinois**	0.8	1.1	1.1	1.5	1.6	2.6	1.8	2.2	**1.6**
**Indiana**	0.7	0.9	1.0	1.2	1.0	1.5	1.5	1.5	**1.2**
**Iowa**	2.8	2.5	2.2	2.3	3.0	4.9	3.5	4.2	**3.2**
**Kentucky**	0.1	0.0	0.1	0.1	0.2	0.4	0.2	0.3	**0.2**
**Michigan**	0.8	0.8	0.8	0.9	0.8	1.2	0.9	1.3	**0.9**
**North Carolina**	0.2	0.2	0.2	0.2	0.3	0.4	0.3	0.4	**0.3**
**North Dakota**	1.2	1.5	3.1	3.2	1.4	1.7	0.3	2.0	**1.8**
**Ohio**	0.3	0.4	0.2	0.3	0.4	0.6	0.8	1.0	**0.5**
**South Dakota**	0.4	0.1	0.1	0.2	0.5	0.4	0.2	0.6	**0.3**
**Tennessee**	0.1	0.2	0.1	0.1	0.0	0.2	0.1	0.1	**0.1**
**West Virginia**	6.5	7.7	6.9	5.8	4.4	6.3	6.1	13.2	**7.1**
Low incidence									
**Alabama**	0.1	0.1	0.0	0.2	0.3	0.2	0.6	0.3	**0.2**
**Alaska**	0.9	1.0	1.0	1.2	0.5	1.9	0.7	0.1	**0.9**
**Arizona**	0.0	0.0	0.0	0.1	0.1	0.3	0.2	0.1	**0.1**
**Arkansas**	0.0	0.0	0.0	0.0	0.0	0.0	0.0	0.0	**0.0**
**California**	0.2	0.3	0.3	0.2	0.2	0.2	0.1	0.2	**0.2**
**Colorado**	0.0	0.0	0.0	0.0	0.0	0.0	0.0	0.0	**0.0**
**Florida**	0.4	0.4	0.3	0.4	0.3	0.4	0.4	0.6	**0.4**
**Georgia**	0.4	0.4	0.1	0.3	0.3	0.1	0.0	0.1	**0.2**
**Hawaii**	0.0	0.0	0.0	0.0	0.0	0.0	0.0	0.0	**0.0**
**Idaho**	0.3	0.3	0.4	0.2	0.0	0.9	0.5	0.2	**0.4**
**Kansas**	0.6	0.6	0.2	0.4	0.3	0.6	0.4	0.4	**0.4**
**Louisiana**	0.1	0.0	0.0	0.0	0.1	0.0	0.0	0.0	**0.0**
**Mississippi**	0.0	0.0	0.0	0.1	0.0	0.0	0.1	0.1	**0.1**
**Missouri**	0.1	0.1	0.1	0.1	0.0	0.0	0.1	0.0	**0.1**
**Montana**	0.6	0.3	0.3	0.9	0.6	1.6	0.5	0.2	**0.6**
**Nebraska**	0.4	0.2	0.4	0.4	0.3	0.4	0.3	0.3	**0.3**
**Nevada**	0.3	0.4	0.1	0.1	0.4	0.4	0.1	0.2	**0.2**
**New Mexico**	0.2	0.0	0.1	0.1	0.0	0.0	0.0	0.0	**0.1**
**Oklahoma**	0.0	0.1	0.0	0.1	0.0	0.0	0.0	0.0	**0.0**
**Oregon**	0.5	0.3	0.2	0.2	0.1	0.3	0.1	0.1	**0.2**
**South Carolina**	0.3	0.5	0.4	0.5	0.7	0.7	0.4	0.3	**0.5**
**Texas**	0.4	0.4	0.2	0.1	0.1	0.2	0.1	0.1	**0.2**
**Utah**	0.1	0.2	0.1	0.2	0.1	0.3	0.2	0.1	**0.2**
**Washington**	0.3	0.2	0.2	0.2	0.2	0.2	0.1	0.2	**0.2**
**Wyoming**	0.2	0.2	0.0	0.2	0.5	0.2	0.3	0.0	**0.3**

**Abbreviation:** N/A = not applicable, not a reportable condition.

* Annual incidence rates per 100,000 population were calculated by state using U.S. Census Bureau estimates from July 1 of each year. U.S. Census Bureau Intercensal Estimates (2000–2010) were used to calculate 2008 and 2009 incidence and Vintage 2015 was used to calculate 2010–2015 incidence.

^†^ State surveillance categories were determined using three classifications: high incidence, neighboring, and low incidence. States with an average annual incidence ≥10 confirmed Lyme disease cases per 100,000 population were classified as high incidence, states sharing a border with those states or located between states with high incidence were classified as neighboring, and all other states were classified as low incidence.

Eleven states and the District of Columbia were classified as neighboring states (Illinois, Indiana, Iowa, Kentucky, Michigan, North Carolina, North Dakota, Ohio, South Dakota, Tennessee, and West Virginia) (Figure 2). During 2008–2015, confirmed cases accounted for 71.0% of the total cases reported from neighboring states. The overall median annual percentage change in number of confirmed cases reported by neighboring states was 6.6% (range: -16.7% to 31.3%). In contrast to states with high incidence, the majority (eight of 11) of neighboring states displayed an overall increasing trend in the number of confirmed cases reported. Among the remaining 25 states, all classified as states with low incidence, confirmed cases accounted for a lower percentage of total cases reported (62.5%).

### Seasonality

**FIGURE 3:**
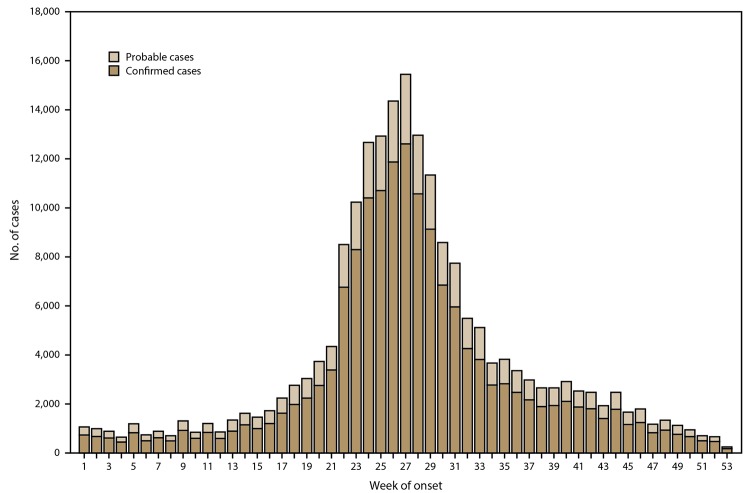
Number* of reported Lyme disease cases, by week of illness onset^†^— United States, 2008–2015 * N = 200,108. ^†^ Week of illness onset was calculated with each week beginning on Sunday. Week 1 of a year was the first week of the year that had at least 4 days in the calendar year; therefore, weeks 1 and 53 sometimes contained days from the preceding or subsequent year.

Information on date of illness onset was available for 200,108 (72.6%) cases. For all years, the first week in July was the peak week of illness onset for confirmed and probable cases (Figure 3).

### Demographics

Information on sex was available for 269,973 (98.0%) cases (97.7% of confirmed cases; 98.7% of probable cases); a majority of cases was among males (56.7%) (Table 2). Information on race was available for 62.1% of cases (129,129 confirmed; 41,883 probable). Most confirmed and probable cases were among white patients (89.7%), followed by other race (6.8%), black (1.6%), Asian/Pacific Islander (1.5%), and American Indian/Alaska Native (<1.0%) patients. A smaller number of case records included information on ethnicity (114,465; 41.5%); of these, 95.9% occurred among non-Hispanic patients.

**TABLE 2:** Demographic characteristics of patients with reported Lyme disease, by case classification and state surveillance category* — United States, 2008–2015

Variable^†^	Confirmed				Probable				Total		
	High incidence	Neighboring	Low incidence	Total confirmed	High incidence	Neighboring	Low incidence	Total probable	High incidence	Neighboring	Low incidence
Sex (% male)	57.2	58.3	47.1	**57.1**	56.2	47.9	41.0	**55.5**	**57.0**	**55.3**	**44.8**
Modal age (yrs)	8	6	51	**8**	56	52	36	**56**	**8**	**9**	**51**
Median age (yrs)	44	39	41	**44**	46	39	41	**45**	**44**	**39**	**41**

* State surveillance categories were determined using three classifications: high incidence, neighboring, and low incidence. States with an average annual incidence ≥10 confirmed Lyme disease cases per 100,000 population were classified as high incidence, states sharing a border with those states or located between states with high incidence were classified as neighboring, and all other states were classified as low incidence.

^†^ Sex: N = 269,973; age: N = 246,840.

**FIGURE 4:**
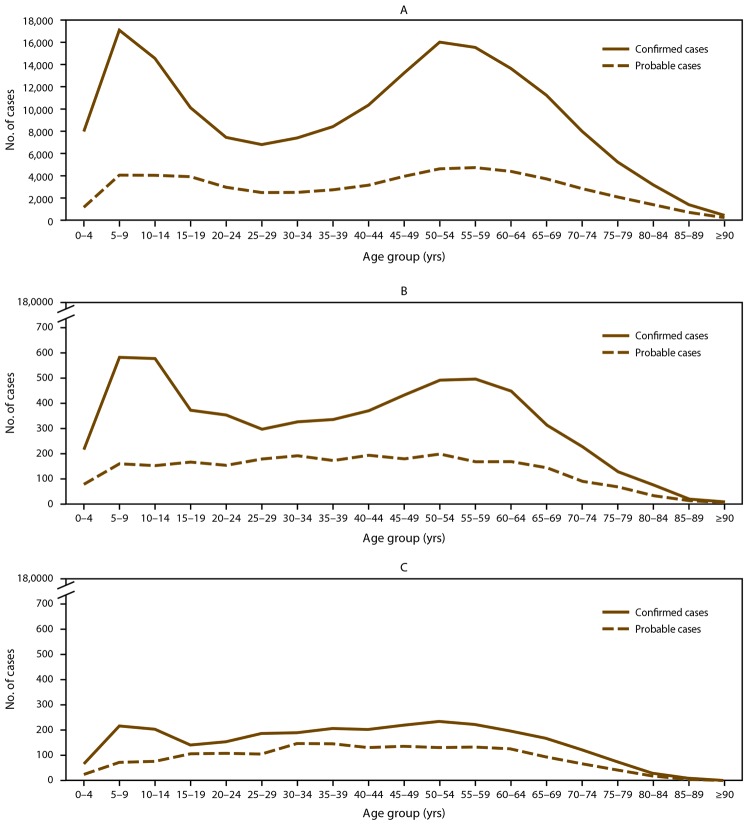
Number* of Lyme disease cases in states with high incidence (A), neighboring states (B), and states with low incidence (C), by age group — United States, 2008–2015 * Number of cases represented in each panel differs according to relative frequency of cases in each state surveillance category (A: high incidence, N = 233,750; B: neighboring states, N = 8,500; C: low incidence, N = 4,590). States with an average annual incidence ≥10 confirmed Lyme disease cases per 100,000 population were classified as high incidence, states that share a border with those states or are located between states with high incidence were classified as neighboring, and all other states were classified as low incidence.

Patient age was available for 246,840 (89.6%) records. The age distribution of patients with confirmed and probable Lyme disease was bimodal with peaks among those aged 5–9 years and 50–55 years (Figure 4). Overall modal age was 8 years, whereas modal age was 8 years among patients with confirmed Lyme disease and 56 years among patients with probable Lyme disease (Table 2).

Demographic characteristics differed among cases reported from states with high incidence, neighboring states, and states with low incidence (Figure 4) (Table 2). In states with high incidence, overall modal age was 8 years, and males accounted for the majority of patients (146,380; 57.0%). In contrast, overall modal age in states with low incidence was 51 years, and males accounted for 44.8% (2,058) of patients (Table 2). In neighboring states, the proportional distribution among males and females was similar to that of states with high incidence; however, modal age was slightly older (9 years) (Table 2).

### Clinical Manifestations

Information on at least one defined clinical manifestation was available for 60.2% of confirmed cases from 35 states. Approximately three fourths (72.2%) of patients had erythema migrans; 27.5% had arthritis; and 1.5% had carditis, defined for surveillance purposes as acute second- or third-degree atrioventricular block. Approximately 12.5% had a neurologic manifestation: 8.4% had facial palsy, 3.8% had radiculoneuropathy, 1.3% had lymphocytic meningitis, and <1.0% had encephalitis. Although the proportion of confirmed case records with indication of erythema migrans was similar between states with high incidence and neighboring states (72.3% [88,090] and 70.6% [2,089], respectively), a lower proportion of records from states with low incidence indicated erythema migrans (581; 64.7%). Neurologic manifestations were more common among patients from neighboring states (731; 24.7%) and states with low incidence (180; 20.0%) compared with states with high incidence (14,823; 12.2%). The proportion of case records with indication of carditis was consistent among all state surveillance categories.

#### Differences Among Demographic Groups

**FIGURE 5:**
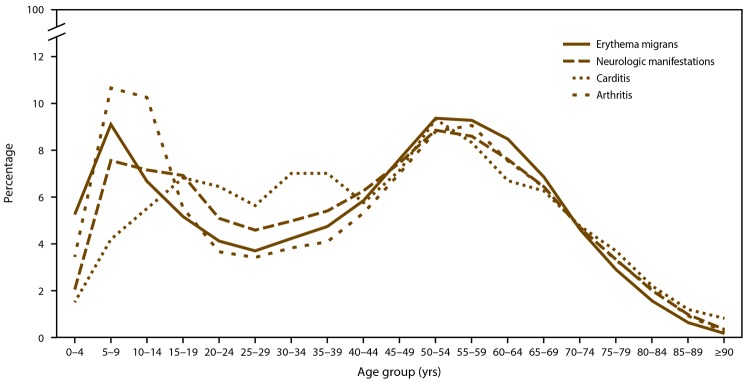
Age distribution of patients with erythema migrans, neurologic manifestations, carditis, and arthritis*^,†^ — United States, 2008–2015 * N = 107,272. ^†^ Age distribution among 90,760 patients with reported erythema migrans, 15,734 patients with reported neurologic manifestations, 1,825 patients with reported carditis, and 37,636 patients with reported arthritis.

Among different clinical manifestations, distribution of confirmed cases varied with respect to patient age but appeared similar among patients in older age groups (>44 years) (Figure 5). Age distributions among patients with erythema migrans and arthritis were bimodal. In contrast, age distributions among patients with carditis and neurologic manifestations were more uniform but still peaked among patients aged 50–55 years (Figure 5). Carditis disproportionately affected patients aged 20–40 years. Although erythema migrans was the most commonly reported clinical sign among all age groups, it was least frequently reported among patients aged 10–14 years (5,143; 60.6%), whereas across all age groups, arthritis was most common among patients aged 10–14 years (2,992; 35.2%). The proportion of male patients with erythema migrans was consistent with the overall sex distribution (50,464; 56.0%); however, among those with carditis, 70.3% (1,279) were male. In addition, black patients comprised a larger proportion of reported cases with carditis (27; 2.3%) when compared with the overall frequency of black patients in the data set (Table 3).

**TABLE 3:** Clinical manifestations of confirmed Lyme disease cases, by patient sex and race — United States, 2008–2015

Characteristic	Arthritis	Erythema migrans	Carditis	Neurologic manifestations
	No. (%)	No. (%)	No. (%)	No. (%)
Sex*				
Male	20,800 (60.2)	50,464 (56.0)	1,279 (70.3)	9,044 (57.9)
Female	13,749 (39.8)	39,671 (44.0)	540 (29.7)	6,589 (42.1)
**Race^†^**				
Native American/Alaska Native	209 (0.9)	226 (0.4)	9 (0.8)	52 (0.5)
Asian/Pacific Islander	340 (1.5)	645 (1.0)	15 (1.2)	137 (1.3)
Black	398 (1.7)	436 (0.7)	27 (2.3)	174 (1.7)
White	20,174 (86.6)	55,847 (90.2)	1,082 (90.0)	9,491 (90.5)
Other	2,175 (9.3)	4,754 (7.7)	69 (5.7)	634 (6.0)

* N = 125,006.

^†^ N = 85,197.

#### Differences in Seasonality

**FIGURE 6:**
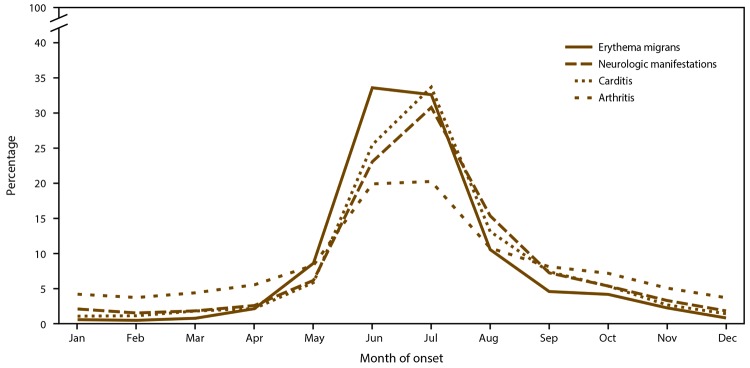
Seasonality of erythema migrans, neurologic manifestations, carditis, and arthritis among confirmed cases of Lyme disease,* by month of onset — United States, 2008–2015 * N = 99,219.

The spring and summer seasonal peak of illness onset among confirmed cases was consistent across clinical manifestations (Figure 6). Erythema migrans was the most commonly reported clinical sign among patients with illness onset during April–November, followed by arthritis (data not shown). Among patients with illness onset during the coldest months (December–March), arthritis was the most common clinical sign.

## Discussion

This report updates available information on the epidemiology of reported Lyme disease cases. For many states with high incidence, the number of case reports appears to have stabilized or declined recently. The decrease in reported cases among many states with high incidence could be attributable to several different factors, including actual stabilization of disease incidence or an artifact from changes in case verification practices designed to minimize the resource demands of conducting Lyme disease surveillance. In contrast, during 2008–2015 the number of cases reported from many of the neighboring states increased. Geographic expansion of areas with substantial occurrence of human Lyme disease is supported by a documented increase in the number of counties in the United States with established *I. scapularis* tick populations (*13*,*14*).

Although the overall demographic and clinical characteristics among reported cases are similar to those detailed in previous reports (*7**,**15*), this report reveals distinct differences in the demographics associated with confirmed and probable cases from states in all surveillance categories*.* Confirmed cases in states with high incidence and neighboring states occurred most commonly among males and with a modal age in young children. In contrast, confirmed cases from states with low incidence were associated with a substantially higher modal age and occurred more commonly among females. Overall, probable cases reflected an older patient population than that of confirmed cases. Although probable cases from states with high incidence still occurred more commonly among males, probable cases from neighboring states and states with low incidence occurred more commonly among females. Demographic differences among cases reported from states with high and low incidence have been previously documented (*16*,*17*) and might reflect lack of specificity of erythema migrans in locations where southern tick-associated rash illness occurs (*18*) as well as higher potential for false positive serologic results stemming from lower positive predictive value of those tests in settings with low incidence (*19*,*20*). Many confirmed cases in states with low incidence likely reflect travel to states with high incidence (*16*)*.* In contrast, probable cases reported from neighboring states and states with low incidence appear to reflect a different patient population, thereby suggesting decreased specificity of the probable case definition in those states.

Approximately 75% of all confirmed case reports that included clinical data had indication of erythema migrans. Infections with illness onset outside the peak season of transmission (spring and summer) might sometimes be considered a result of infection that was acquired during the spring and summer months but did not clinically manifest until months later. Arthritis, the most common disseminated manifestation of Lyme disease, was the most common clinical finding among patients with reported illness onset during the coldest months in the temperate United States. Nevertheless, erythema migrans was the most commonly reported sign of infection among patients with illness onset not only in the peak spring and summer months but for two thirds of the year (April–November) (data not shown). This seasonal pattern underscores that adult ticks that seek blood meal hosts during the fall months have a proportional role in human illness and that prevention messages should not be focused only during the spring and summer season when nymphal ticks seek hosts.

The usefulness of Lyme disease surveillance differs across jurisdictions. High numbers of possible Lyme disease cases that require clinical follow-up have taxed public health resources in states with high incidence to an unsustainable level (*21*). Solutions vary, with some states investigating cases as resources allow, which at times means curtailing surveillance activities. Other states have begun to employ statistical methodology to estimate the number of cases each year. For example, several counties in New York have implemented a system in which 20% of positive laboratory reports are sampled and investigated to determine what proportion can be confirmed; these results are extrapolated to the remaining unsampled laboratory reports to arrive at an estimate of Lyme disease case counts in those counties (*22*). Several states are considering adopting similar methodologies to better manage public health surveillance for Lyme disease (*23*). In line with historical case-based surveillance systems, case estimates are not reported to CDC through NNDSS, one of several factors that contribute to underreporting of cases nationally. In areas where Lyme disease incidence has remained high for years, expensive, ongoing surveillance does not yield new information about the magnitude or geographic distribution of the disease and potentially diverts limited public health resources that might be spent on prevention. Taken together, these points suggest the need for a paradigm shift in states with high incidence that would minimize personnel and resource costs while still maintaining awareness of the disease. In contrast, public health surveillance in states where Lyme disease is emerging can serve to increase knowledge of local disease incidence and spread, which can in turn be used to target educational measures for health care providers and the public.

To improve specificity of reported cases in areas with low incidence and areas where Lyme disease is emerging, CSTE voted to modify the Lyme disease surveillance case definition effective in 2017 (*24*). Confirmation of infection acquired in states outside those with high incidence now requires laboratory evidence of infection. As Lyme disease emerges in neighboring states, clinical suspicion of Lyme disease in a patient should be based on local experience rather than incidence cutoffs used for surveillance purposes.

Identification of effective methods to prevent Lyme disease has proven challenging. Measures aimed at reducing tick populations on residential properties have not proven effective in decreasing the number of human Lyme disease infections (*25*). Long recommended behavioral interventions, such as wearing permethrin-treated clothing or using repellent containing DEET, have not been adequate to control Lyme disease on a population scale (*26*,*27*). In addition, adherence to recommendations aimed at preventing Lyme disease has been poor, even in areas of high risk (*28*,*29*). New approaches are needed to reduce the incidence and spread of Lyme disease, including exploration of a second-generation human vaccine (*28*)*.*

## Limitations

Because systematic interpretation of Lyme disease surveillance data has been and continues to be complicated by several factors, this report is subject to at least three limitations. First, recent estimates of underreporting to the public health system suggest that the actual incidence in the United States might be tenfold higher than final reported cases (*30*–*32*). At the same time, Lyme disease is subject to misdiagnosis (specifically false positive diagnosis), especially in areas where the disease is rare and false positive test results are more likely (*32*). Shifts in annual case counts in a state might not reflect actual changes in disease incidence but might often be linked to changes in surveillance practices within that state or to competing public health priorities, such as the H1N1 influenza epidemic or the 2014 Ebola outbreak in West Africa, that tap limited public health personnel. Because of the resources required to conduct Lyme disease surveillance, many jurisdictions with high incidence have implemented modifications to methods of case ascertainment and verification. The resulting heterogeneous nature of surveillance data limits interpretability over time and across jurisdictions. Second, the classification of neighboring state used for this report was based on administrative boundaries, and these states do not exhibit uniform risk for Lyme disease or patterns of associated disease emergence. For example, although Iowa, Illinois, Kentucky, and Tennessee are all classified as neighboring states in this analysis, risk for Lyme disease is clear in specific areas of Iowa and Illinois but negligible in Kentucky and Tennessee, which have limited numbers of infected host-seeking vector ticks. Furthermore, Lyme disease is endemic in certain areas of the Pacific Coast that support the enzootic cycle, and although risk is documented in those areas, no states outside of the Northeast, mid-Atlantic, or upper Midwest regions met the criteria for high incidence. Finally, in many states with low incidence, cases likely reflect travel of persons and acquisition of infection in states with high incidence rather than local transmission (*16*)*.*

## Conclusion

This summary provides an updated description of the epidemiology of Lyme disease in the United States. During 2008–2015, similar to previous periods, the number of Lyme disease cases fluctuated from year to year; however, the total number of reported cases remained above 30,000 each year, making Lyme disease the most commonly reported vectorborne disease in the country (*12*,*33*). Overall, regions with highest risk for Lyme disease and populations in which most cases occur remain similar to those previously described (*7*), although expansion into neighboring states is evident. Reported cases exhibited a bimodal age distribution and occurred more commonly among males and during the early summer months when the nymphal stage *Ixodes* spp. vector ticks are seeking blood meal hosts in North America. Probable cases display more uniform age and sex distributions than confirmed cases. Unlike the predominance among males and a bimodal age distribution apparent when looking at trends among all cases, cases in states with low incidence are more common among women aged 15–59 years.

Lyme disease surveillance is not meant to document every case but rather to indicate disease trends over time, define high-risk groups, and describe the geographic distribution of the condition. Lyme disease surveillance is challenging, and Lyme disease continues to present a major public health problem in multiple regions of the United States.
